# Melatonin and self harm behavior in youth, beyond the sleep impact

**DOI:** 10.1192/j.eurpsy.2024.962

**Published:** 2024-08-27

**Authors:** N. Kouki, A. Maamri, N. kouki, F. Ghrissi, A. Hajri, H. zalila

**Affiliations:** Outpatient psychiatry department, razi, manouba, Tunisia

## Abstract

**Introduction:**

Sleep disorders in youth are associated to psychiatric disorders and may lead to significant negative effects on cognitive skills, emotional regulation and behavior such as self harm.

**Objectives:**

The aim of our study is to highlight the melatonin effects on reducing self harm behaviors in the youth.

**Methods:**

Our work is a literature review based on the PubMed interface and adapted for 2 databases: Science Direct and Google Scholar using the following combination ( self harm [MeSH terms]) AND ( melatonin [MeSH terms]) AND ( youth [MeSH terms]) .

**Results:**

We initially reviewed 6 articles published between 2012 and 2022. We retained 3 articles which corresponded to the aim of our study.

Self harm behaviors were mainly described in youth during adolescenthood. In fact, self injurious release may be considered as way to release emotional tension and physical discomfort.

Melatonin prescribed in youth for the treatment of sleep disorders not only improved sleep ,but also mood disorders and impulsivity. Melatonin restores indirectly serotonin levels through a continuous bidirectional connexion . Therefore it is efficient on psychiatric comorbidites, especially anxiety and depression which are associated with intentional self-harm.

**Conclusions:**

Melatonin is the most prescribed drug for sleep disturbances in children and adolescents, its impact covers a large spectrum of disturbances including the self harm behaviors.

**Disclosure of Interest:**

None Declared

